# Women in chemistry: Q&A with Professor Hyunjoo Lee

**DOI:** 10.1038/s42004-024-01291-3

**Published:** 2024-09-25

**Keywords:** Catalyst synthesis

## Abstract

Prof. Hyunjoo Lee is a Full Professor in the Department of Chemical and Biomolecular Engineering at the Korea Advanced Institute of Science & Technology (KAIST), Korea, and a KAIST Endowed Chair Professor. She also serves as the Director of the Heterogeneous Atomic Catalysts Research Center.

**Figure:**
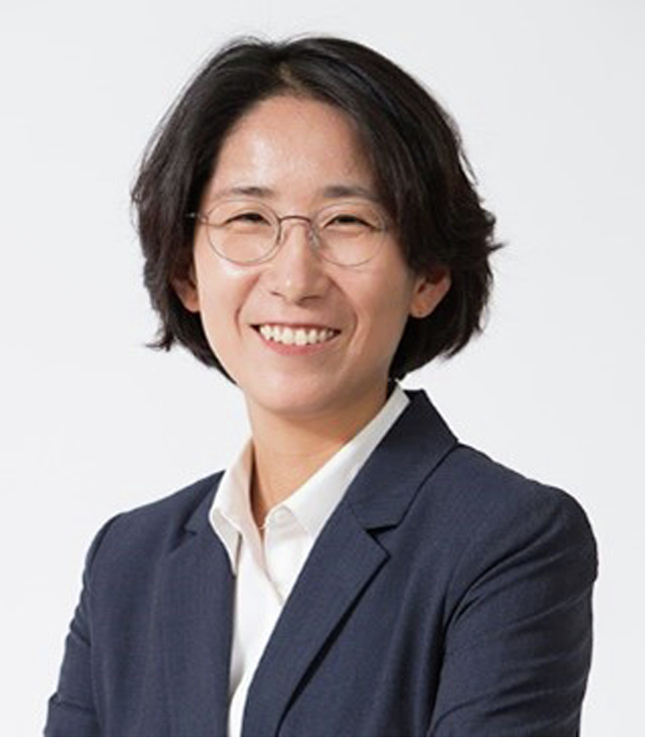
Prof. Hyunjoo Lee

Prof. Lee earned her Ph.D. in Chemical Engineering from Caltech in 2005. Her research is at the forefront of heterogeneous catalysis, focusing on unraveling the fundamental mechanisms of catalytic surfaces and developing advanced catalysts for sustainable chemical processes. She has published approximately 160 papers aimed at elucidating how the surfaces of heterogeneous catalysts can be controlled to improve activity, selectivity, and durability. Prof. Lee holds around 50 patents in areas including fuel cells, water and CO_2_ electrolyzers, automobile exhaust treatment, and biomass conversion. She is currently an Associate Editor of *JACS Au* and serves on the editorial boards of *ChemSusChem, Nano Letters, Molecular Catalysis, Catalysis Today, ACS Applied Energy Materials*, and other leading journals.

Why did you choose to be a scientist?

When I was young, I was captivated by the science magazine *Newton*, particularly the articles on astronomy and Earth sciences. I was always in awe of how humans, living on a small planet for such a brief time, could uncover the mysteries of space, spanning millions of light-years. This deepened my love and admiration for the power of science, which has brought about remarkable advancements such as engines, airplanes, fertilizers, and telecommunications. When the time came to choose a major, I was driven by a desire to contribute to environmental protection, and I realized that chemical engineering offered the perfect avenue to pursue that goal.

What scientific development are you currently most excited about?

I’m most excited about our work in developing heterogeneous ‘atomic’ catalysts. Our focus is on precisely controlling the atomic configuration of surface-active sites. For example, we’ve synthesized platinum (Pt) single atoms on a titanium nitride (TiN) support for the electrochemical oxygen reduction reaction, and Pt ensembles on a ceria-alumina support for three-way catalytic reactions. Although heterogeneous catalysts are widely used in industry, understanding their mechanisms and achieving precise structural control has remained challenging. By leveraging single-atom catalysts, we are meticulously engineering surface structures to maximize activity, selectivity, and durability. Recently, we succeeded in elucidating the oxygen transfer behavior on ceria with Pt single atoms, effectively isolating the role of ceria in the oxidation reaction, independent of Pt sites. This breakthrough allows us to better understand and harness the contributions of ceria in catalytic processes.

What direction do you think your research field should go in?

The chemical industry has undergone rapid changes over the past decade. Traditionally, various chemicals and fuels were produced from petroleum, but we are now striving to create these from sustainable resources such as biomass or CO_2_. While large-scale chemical plants were previously built to produce these products cost-effectively, future chemical plants will be much smaller and will utilize a diverse range of raw materials and energy sources. High-performance catalysts will be key to realizing this significant transition. My goal is to develop catalysts with high activity, selectivity, and durability for the production of energy and chemicals, which can be easily adapted to the small-scale, innovative chemical plants of the future.

Have you been a minority as a woman at any stage of your career? What was that experience like for you?

I grew up in a family with a strong patriarchal culture. My parents often told me that I should study hard to set a good example for my younger brother, but they never envisioned an independent and successful career for their intelligent daughter. This reflected the typical social atmosphere in which I was raised. When I entered Seoul National University, one of South Korea’s top universities, there were only five female students out of 75 in my department. Even more discouraging, there were no female faculty members in the entire College of Engineering. At that time, I couldn’t envision what my future would look like if I continued pursuing my career, which led me to pursue my Ph.D. in the United States. Later, I realized that pursuing an academic career as a woman is challenging, even in America, but I was fortunate to meet many people who encouraged and supported me along the way.

How can young women in the field be supported to become established scientists?

South Korea’s social landscape has changed rapidly over the past few decades. While the percentage of female students in engineering was very low 30 years ago, today, women make up about 30% of the students in the Department of Chemical Engineering. As part of the first generation of female engineering faculty, I’ve seen a significant increase in the number of female professors among the younger generation. The concerted efforts by the government and universities in South Korea to hire more female professors have been highly effective. Female college students now have visible role models in their schools, enabling them to pursue careers in engineering with confidence. Our current focus is on strengthening social support systems for babies and young children, which is crucial for balancing career aspirations with family life.

Are you or have you been supported by a mentor? What was the best advice you received?

I have been fortunate to have had exceptional advisors throughout my academic education. Prof. Jongheop Yi at Seoul National University (Master advisor), Prof. Mark E. Davis at Caltech (Ph.D. advisor), and Prof. Peidong Yang at UC Berkeley (Post-doc. advisor) have all been incredibly supportive, and I have learned immensely from each of them. I am especially grateful for Mark’s advice: ‘Ask the right question’ and ‘Keep fighting to be excellent.’ I have come to realize that excellent research begins with asking the right questions. As an introverted Asian female, I have often tended to hold back, trying to remain humble and polite. From Mark, I learned the importance of fighting to demonstrate the excellence of my work.

Do you have any advice you would like to share with women starting out in chemical research?

My primary advice is to prioritize excellence in your work. Never compromise on your professional competence, no matter the circumstances. Along the way, you may encounter frustrations in your scientific work or in your relationships with colleagues, but it is crucial to protect your physical and mental health. Building a strong network in your field is also essential. As you embark on your independent career, your reputation will be critical for securing research funding and publishing your work in prestigious journals. Even if you are an excellent scientist, if people in the field aren’t familiar with your work, your papers and proposals may be undervalued. I hope to see more female colleagues in chemistry and chemical engineering who are not only highly capable in their work but also fulfilled in their personal lives.

*This interview was conducted by the editors of Communications Chemistry*.

